# Bevacizumab or Aflibercept in Combination with Second-Line FOLFIRI after Progression on FOLFOX Plus Bevacizumab in Metastatic Colorectal Cancer: A Comparative Study

**DOI:** 10.7150/jca.138530

**Published:** 2026-09-12

**Authors:** Okan Aydin, Sermin Dinc Sonusen, Asli Buyukkuscu, Seyyit Bayram Sahin, Serhat Kalkan, Ahmet Emin Ozturk, Mert Erciyestepe, Fatih Atalah, Zehra Sucuoglu Isleyen, Emir Celik, Kayhan Erturk, Muhammed Mustafa Atci

**Affiliations:** 1 Department of Medical Oncology, University of Health Sciences, Prof. Dr. Cemil Tascioglu City Hospital, Istanbul, Turkey.; 2 Department of Medical Oncology, Istinye University Faculty of Medicine, Liv Hospital, Istanbul, Turkey.

**Keywords:** metastatic colorectal cancer, FOLFIRI, bevacizumab, aflibercept, second-line therapy

## Abstract

**Background:**

The optimal anti-angiogenic strategy in the second-line treatment of metastatic colorectal cancer (mCRC) after progression on first-line FOLFOX plus bevacizumab remains unclear. We aimed to compare the efficacy and safety of FOLFIRI combined with bevacizumab or aflibercept in this well-defined clinical setting.

**Methods:**

In this retrospective single-center study, 152 patients with mCRC who progressed after at least four cycles of first-line FOLFOX plus bevacizumab were included. Of these, 92 patients received FOLFIRI plus bevacizumab and 60 received FOLFIRI plus aflibercept as second-line therapy. Stabilized inverse probability of treatment weighting (IPTW) was applied to account for baseline differences between treatment groups. PFS and OS were evaluated using IPTW-weighted Kaplan-Meier and Cox regression analyses.

**Results:**

No statistically significant difference was observed between the bevacizumab and aflibercept groups for PFS (HR: 1.302, 95% CI: 0.929-1.825, p=0.125) or OS (HR: 1.212, 95% CI: 0.846-1.736, p=0.295) in the IPTW-weighted analyses. Treatment type was not independently associated with PFS or OS in multivariate analyses. Grade 3-4 toxicities were observed in both groups, with neutropenia being the most common; thrombocytopenia and neutropenia were numerically more frequent in the aflibercept group.

**Conclusion:**

No statistically significant differences in efficacy were observed between FOLFIRI plus bevacizumab and FOLFIRI plus aflibercept in patients with mCRC progressing after first-line FOLFOX plus bevacizumab. Both strategies represent reasonable second-line options in this setting, highlighting the need for prospective studies to define the optimal sequencing of anti-angiogenic therapies.

## Introduction

Colorectal cancer (CRC) represents a major global health burden, ranking as the third most commonly diagnosed cancer and the second leading cause of cancer-related mortality worldwide, with approximately 1.9 million new cases and 900,000 deaths reported in 2022 (GLOBOCAN) 1. Prognosis in colorectal cancer is largely determined by the stage at diagnosis. Approximately 25% of patients present with metastatic disease, while a substantial proportion of those initially diagnosed with localized tumors eventually develop metastases during follow-up 2,3. Despite advances in treatment, outcomes in metastatic colorectal cancer remain poor, with 5-year overall survival rates of approximately 15% 3.

Systemic therapy forms the backbone of treatment in metastatic colorectal cancer (mCRC), primarily consisting of fluoropyrimidines (5-fluorouracil or capecitabine) in combination with oxaliplatin or irinotecan. Doublet chemotherapy regimens such as FOLFOX, CAPOX (XELOX), and FOLFIRI are commonly used in the first-line setting, while triplet regimens (FOLFOXIRI) may be considered in selected patients 4-6. In addition to cytotoxic chemotherapy, the incorporation of targeted biological agents has significantly improved clinical outcomes. These include monoclonal antibodies directed against the epidermal growth factor receptor (EGFR), such as cetuximab and panitumumab, which are restricted to RAS wild-type tumors, and anti-angiogenic agents targeting the vascular endothelial growth factor (VEGF) pathway, including bevacizumab and aflibercept. The addition of these targeted therapies to chemotherapy has been shown to improve both progression-free and overall survival in patients with mCRC 7,8.

The selection of treatment for metastatic colorectal cancer is increasingly guided by molecular and clinical characteristics, including the location of the primary tumor and its mutational status. Tumors arising in the right and left colon exhibit distinct biological behaviors and respond differently to targeted therapies. Anti-EGFR agents, for example, are primarily effective in patients with left-sided RAS wild-type tumors, whereas their benefit is limited in those with RAS or BRAF mutations. Given that RAS mutations are present in approximately half of patients and BRAF mutations in a smaller subset, anti-angiogenic strategies play a central role in a substantial proportion of patients 9,10. In this context, bevacizumab, a monoclonal antibody that targets the VEGF pathway, is commonly used alongside chemotherapy as a first-line treatment. It has been demonstrated to enhance both progression-free and overall survival, regardless of tumor location or RAS mutation status 11,12.

In patients with metastatic colorectal cancer who experience disease progression following first-line oxaliplatin-based chemotherapy combined with bevacizumab, switching to an irinotecan-based regimen such as FOLFIRI represents the standard second-line approach 13. In this setting, continuation of anti-angiogenic therapy beyond progression has been shown to provide additional clinical benefit. Bevacizumab may be maintained in combination with second-line chemotherapy, as demonstrated in randomized studies showing improved survival outcomes compared with chemotherapy alone 14. Alternatively, other anti-angiogenic agents such as aflibercept can be combined with FOLFIRI after progression on oxaliplatin-based regimens 15,16. Accordingly, evidence from phase III trials supports the use of both bevacizumab and aflibercept as effective anti-angiogenic options in the second-line treatment of mCRC following progression on bevacizumab-containing first-line therapy. However, no randomized head-to-head trial has directly compared these two anti-VEGF agents in the second-line setting.

Despite the availability of both bevacizumab and aflibercept in the second-line setting, the optimal choice between these agents remains unclear. Although multicenter real-world data are available, additional evidence from strictly defined treatment-sequence cohorts may help clarify the comparative effectiveness of these strategies in routine clinical practice. Therefore, this study aimed to compare the efficacy and safety of second-line FOLFIRI plus bevacizumab versus FOLFIRI plus aflibercept in patients with metastatic colorectal cancer who experienced disease progression following first-line FOLFOX plus bevacizumab.

## Materials and Methods

This retrospective observational study screened patients with metastatic colorectal cancer (mCRC) who received first-line FOLFOX plus bevacizumab at our institution between January 2015 and December 2025. Patients who received at least four cycles of first-line FOLFOX plus bevacizumab and had at least one radiological response assessment were considered eligible. Those who developed disease progression were included in the analysis. A total of 152 patients met these criteria. Of these, 60 patients received FOLFIRI plus aflibercept and 92 patients received FOLFIRI plus bevacizumab as second-line treatment. This study reflects the single-center experience of our institution.

Patients were excluded if they were younger than 18 years, did not develop disease progression following first-line treatment, had not received first-line FOLFOX plus bevacizumab, or did not receive second-line FOLFIRI combined with either bevacizumab or aflibercept. Clinical, pathological, and laboratory data were retrospectively obtained from hospital records.

Second-line treatment consisted of FOLFIRI in combination with either bevacizumab or aflibercept. Bevacizumab (5 mg/kg, every 2 weeks) or aflibercept (4 mg/kg, every 2 weeks) was administered in combination with FOLFIRI until disease progression or unacceptable toxicity. Patients who received at least four cycles of second-line treatment and had at least one radiological response assessment were included in the survival analysis. A total of 60 patients treated with FOLFIRI plus aflibercept and 92 patients treated with FOLFIRI plus bevacizumab were compared in terms of overall survival (OS) and progression-free survival (PFS). Safety and treatment-related adverse events were also evaluated.

Overall survival (OS) was defined as the time from the initiation of second-line treatment to death from any cause or last follow-up. Progression-free survival (PFS) was defined as the time from the initiation of second-line treatment to disease progression or death.

Statistical analyses were performed using R software, version 4.5.1. Weighted Kaplan-Meier analyses and weighted Cox proportional hazards models were implemented using the survival package (version 3.8-3). Covariate balance diagnostics were performed using the cobalt package (version 4.6-1), robust covariance estimation using the sandwich package (version 3.1-1), and survival plots were generated using survminer (version 0.5-1) and ggplot2 (version 4.0.0). Categorical variables were expressed as frequencies and percentages, while continuous variables were summarized as means with standard deviations or medians with ranges, as appropriate.

To account for potential baseline confounding between the treatment groups, propensity scores for receiving aflibercept were estimated using binomial logistic regression including sex, age group, Eastern Cooperative Oncology Group (ECOG) performance status, comorbidity, tumor location, microsatellite instability (MSI) status, de novo metastatic presentation, RAS/BRAF mutation status, and the presence of liver, lung, peritoneal, bone, and non-regional lymph-node metastases. Stabilized inverse probability of treatment weighting (IPTW) targeting the average treatment effect (ATE) was then applied. All patients were retained in the IPTW analysis. Covariate balance after weighting was primarily assessed using absolute standardized mean differences (SMDs), with values <0.10 considered indicative of acceptable balance. Weighted p values were reported as supplementary descriptive measures and were not used for covariate selection.

Progression-free survival (PFS) and overall survival (OS) were evaluated using IPTW-weighted Kaplan-Meier estimates and weighted Cox proportional hazards regression models. Confidence intervals and p values from the weighted Cox models were based on robust sandwich standard errors clustered at the patient level. In multivariable Cox regression analyses for PFS and OS, age, sex, and ECOG performance status were included regardless of statistical significance, along with variables showing a p value <0.20 in univariable analysis. A two-sided p value <0.05 was considered statistically significant. As a sensitivity analysis, patients with unknown MSI status were excluded, and treatment effects on PFS and OS were re-estimated with additional adjustment for MSI status to assess the potential impact of missing MSI data.

This retrospective study was approved by the Ethics Committee of the University of Health Sciences, Prof. Dr. Cemil Tascioglu City Hospital, Istanbul, Turkey (Approval No: 2025/277). The study was conducted in accordance with the principles of the Declaration of Helsinki. The requirement for informed consent was waived by the Ethics Committee of the University of Health Sciences, Prof. Dr. Cemil Tascioglu City Hospital, Istanbul, Turkey due to the retrospective nature of the study.

OpenAI ChatGPT was used solely for language editing and to improve the clarity and readability of the text throughout the manuscript. It was not used for data collection, statistical analysis, generation of results, or interpretation of the study findings. All AI-assisted edits were reviewed and approved by the authors.

## Results

A total of 152 patients with mCRC who met the inclusion criteria were included in the study. Of these, 60 patients received FOLFIRI plus aflibercept and 92 patients received FOLFIRI plus bevacizumab as second-line treatment. Baseline demographic and clinicopathological characteristics of the study population, together with covariate balance after IPTW, are summarized in Table 1. The mean age of the study population was 58.7 ± 12.3 years, and the median follow-up duration was 56.4 months.

In both treatment groups, the majority of patients were male, and sex distribution was similar between the groups. All patients had an ECOG performance status of 0-1, indicating a relatively well-preserved functional status. The majority of patients in both groups had at least one comorbidity. The most common of these were hypertension, diabetes mellitus, chronic obstructive pulmonary disease/asthma, and ischemic heart disease. Histologically, the vast majority of tumors were adenocarcinomas (143/152), and primary tumor location was predominantly in the left colon. Most tumors were microsatellite stable (MSS), while microsatellite instability-high (MSI-H) tumors were rare, and a substantial proportion had unknown MSI status. Approximately 90% of patients presented with de novo metastatic disease. Most patients were not eligible for surgical resection, and the distribution of resection status was similar between the two groups. In the metastatic setting, 48 patients underwent surgical resection, of whom only 14 achieved R0 resection, suggesting that most procedures were performed with non-curative intent.

In terms of molecular characteristics, RAS/BRAF mutations were detected in 102 of 152 patients, whereas approximately one-third of patients had no detectable mutation. KRAS mutations were the most frequent among mutated cases, whereas NRAS and BRAF mutations were less common. The liver was the predominant site of metastasis, followed by the lung and peritoneum, whereas bone and non-regional lymph node metastases were relatively uncommon. In the metastatic setting, 22 patients received local treatment, most commonly metastasectomy, whereas radiofrequency ablation, transarterial chemoembolization, and radiotherapy were less frequently used.

Before weighting, baseline imbalances were observed particularly in ECOG performance status and MSI status. Patients in the FOLFIRI plus aflibercept group had a higher proportion of ECOG 0, whereas the bevacizumab group had a higher proportion of MSS tumors. To account for baseline differences between the treatment groups, stabilized inverse probability of treatment weighting (IPTW) was applied. Following IPTW, covariate balance improved substantially, with all baseline covariates achieving a maximum absolute standardized mean difference (Max |SMD|) below 0.10, indicating adequate balance between the treatment groups (Table 1).

In IPTW-weighted univariate Cox regression analysis, older age (≥65 years), liver metastasis, and bone metastasis were significantly associated with shorter PFS (HR: 1.627, 95% CI: 1.158-2.286, p=0.005; HR: 1.513, 95% CI: 1.012-2.263, p=0.043; and HR: 1.778, 95% CI: 1.100-2.875, p=0.019, respectively). Other evaluated clinicopathological and treatment-related variables, including treatment type, were not significantly associated with PFS (Table 2).

In the IPTW-weighted multivariate analysis, older age (≥65 years), liver metastasis, and bone metastasis remained independently associated with shorter PFS (HR: 1.857, 95% CI: 1.226-2.812, p=0.003; HR: 1.758, 95% CI: 1.176-2.629, p=0.006; and HR: 2.208, 95% CI: 1.276-3.820, p=0.005, respectively). Treatment with aflibercept versus bevacizumab was not significantly associated with PFS after adjustment (HR: 1.347, 95% CI: 0.960-1.889, p=0.085). Thus, the IPTW-weighted analysis did not demonstrate a statistically significant difference in PFS according to second-line anti-angiogenic treatment.

In IPTW-weighted univariate Cox regression analysis for OS, bone metastasis was significantly associated with shorter OS (HR: 2.136, 95% CI: 1.225-3.726, p=0.007) (Table 3). Older age (≥65 years) showed a nonsignificant trend toward shorter OS (HR: 1.435, 95% CI: 0.989-2.082, p=0.057). Treatment with aflibercept versus bevacizumab was not significantly associated with OS (HR: 1.212, 95% CI: 0.846-1.736, p=0.295). Other evaluated clinicopathological and treatment-related variables were not significantly associated with OS.

In the IPTW-weighted multivariate analysis, bone metastasis remained independently associated with shorter OS (HR: 2.354, 95% CI: 1.291-4.294, p=0.005). Older age remained nonsignificant (HR: 1.545, 95% CI: 0.941-2.536, p=0.085), and no significant association was observed between treatment type and OS (HR: 1.122, 95% CI: 0.751-1.678, p=0.573). These findings indicate that the absence of a significant difference in OS between the two treatment strategies persisted after IPTW adjustment.

In the IPTW-weighted analysis, the PFS curves of the bevacizumab and aflibercept groups largely overlapped, although PFS appeared numerically more favorable with bevacizumab during parts of the follow-up period. However, no statistically significant difference in PFS was observed between the treatment groups (robust weighted Cox p=0.125) (Figure 1).

In the IPTW-weighted analysis, the OS curves of the bevacizumab and aflibercept groups largely overlapped throughout follow-up, with substantial overlap of the confidence intervals. No statistically significant difference in OS was observed between the treatment groups in the robust weighted Cox analysis (p=0.295) (Figure 2).

To assess the potential impact of missing MSI data, a sensitivity analysis was performed after restricting the cohort to patients with known MSI status (n=101). The treatment effect remained non-significant for both PFS (aflibercept vs bevacizumab: HR 1.342, 95% CI 0.889-2.026, p=0.161) and OS (HR 1.136, 95% CI 0.719-1.797, p=0.585). These findings were consistent with the primary IPTW analyses, suggesting that exclusion of patients with unknown MSI status did not materially alter the comparative treatment-effect estimates.

After stabilized IPTW, grade 3-4 neutropenia was numerically more frequent in the aflibercept group than in the bevacizumab group (20.3% vs 11.5%). Grade 3-4 thrombocytopenia was also numerically more frequent with aflibercept (15.6% vs 3.3%). In contrast, the weighted frequencies of anemia, diarrhea, neuropathy, and nephrotoxicity were generally comparable between the treatment groups. Hepatotoxicity was observed in the bevacizumab group (2.2%) but not in the aflibercept group (Table 4).

At the time of analysis, 7 patients in the FOLFIRI plus bevacizumab group had not experienced disease progression and were continuing maintenance therapy with fluorouracil-folinic acid plus bevacizumab, while 4 patients in the FOLFIRI plus aflibercept group were continuing fluorouracil-folinic acid plus aflibercept. Following progression on second-line treatment, 48 patients in the bevacizumab group and 28 patients in the aflibercept group received third-line therapy. At the time of analysis, 14 patients in the bevacizumab group and 11 patients in the aflibercept group were alive, whereas 78 and 49 patients had died, respectively.

## Discussion

In this retrospective real-world study, we compared the efficacy and safety of FOLFIRI plus bevacizumab and FOLFIRI plus aflibercept as second-line treatment in patients with metastatic colorectal cancer who had progressed after first-line FOLFOX plus bevacizumab. Our results demonstrated that there was no statistically significant difference between the two treatment groups in terms of progression-free survival and overall survival after IPTW adjustment. In the multivariable analyses, older age, liver metastasis, and bone metastasis were independently associated with shorter PFS, while bone metastasis was independently associated with shorter OS. Grade 3-4 treatment-related toxicities were observed in both groups. Neutropenia was the most frequently reported severe adverse event, while neutropenia and thrombocytopenia were numerically more frequent in the aflibercept group. Taken together, these findings suggest that both bevacizumab and aflibercept represent feasible anti-angiogenic options in combination with FOLFIRI in the second-line setting in real-world clinical practice.

Several real-world studies have evaluated the efficacy of FOLFIRI in combination with anti-angiogenic agents as second-line treatment in patients with metastatic colorectal cancer. In a recent study conducted in South Korea, no significant differences were observed between FOLFIRI plus bevacizumab and FOLFIRI plus aflibercept in terms of response rate, progression-free survival, and overall survival. The median progression-free survival and overall survival reported in that study were highly consistent with the findings of our study 17. However, although most patients in that study received first-line FOLFOX plus bevacizumab, a subset in the bevacizumab group had been treated with FOLFOX plus cetuximab. Therefore, unlike our fully homogeneous post-bevacizumab cohort, the study population remains partially heterogeneous, which should be considered when interpreting the results.

A large multicenter retrospective study from France (BEFLICO study) evaluated a highly comparable clinical scenario, including patients who progressed after first-line FOLFOX plus bevacizumab and subsequently received second-line FOLFIRI in combination with either bevacizumab or aflibercept. In contrast to our findings, that study demonstrated a statistically significant improvement in both progression-free survival and overall survival in favor of bevacizumab, even after multivariate adjustment 18. Several factors may explain these discrepant findings. First, differences in molecular characteristics, particularly the higher prevalence of RAS mutations in the BEFLICO cohort, may have influenced treatment response to anti-angiogenic strategies. Second, the substantially larger sample size and multicenter design of the BEFLICO study may have increased its statistical power to detect differences between treatment groups. Finally, inherent limitations of retrospective analyses, including potential selection bias and variability in treatment decision-making, should also be considered when interpreting these results.

In another study evaluating bevacizumab and aflibercept in combination with FOLFIRI in the second-line setting, no significant difference was observed in progression-free survival; however, bevacizumab-based therapy was associated with improved overall survival and a more favorable toxicity profile 19. These findings are consistent with those reported in the BEFLICO study, which suggests that continuing bevacizumab treatment beyond progression may be beneficial. However, while the majority of patients in the BEFLICO study had received bevacizumab as a first-line treatment, some had not. Furthermore, the study population was relatively small and heterogeneous in terms of molecular characteristics, which may limit direct comparability with our more homogeneous cohort.

A similar clinical setting was evaluated in a recent multicenter Turkish study, which included patients with RAS-mutant metastatic colorectal cancer who progressed after first-line oxaliplatin-based chemotherapy combined with bevacizumab and were subsequently treated with FOLFIRI plus either bevacizumab or aflibercept. In contrast to our findings, that study demonstrated significantly improved progression-free survival and overall survival in the bevacizumab group, along with a more favorable toxicity profile 20. These results are consistent with those reported in the BEFLICO study and further support the potential benefit of continuing bevacizumab beyond progression. Taken together, our findings provide complementary real-world evidence from a strictly defined treatment-sequence cohort and suggest that the comparative effectiveness of these strategies may vary across clinical populations.

However, an important distinction is that the Turkish study included only patients with RAS-mutant tumors, whereas our study population was not restricted by RAS status. Given that RAS-mutant tumors are less likely to benefit from anti-EGFR therapies and are more commonly treated with anti-angiogenic strategies, this molecular selection may have influenced treatment outcomes. Therefore, differences in patient selection, particularly with respect to RAS status, may partly explain the discrepancy between our results and those of previous studies.

Another real-world study focusing on RAS-mutant metastatic colorectal cancer, conducted by Ottaiano et al. in Italy, reported comparable survival outcomes between FOLFIRI plus bevacizumab and FOLFIRI plus aflibercept; however, a non-significant trend toward improved survival was observed in the aflibercept group 21. Furthermore, the ongoing ARBITRATION study, a prospective observational trial in patients with RAS-mutant disease progressing after bevacizumab-based first-line therapy, is designed to address this question and may help clarify the conflicting findings of previous retrospective studies 22.

The efficacy of aflibercept in combination with FOLFIRI in the second-line setting was demonstrated in the pivotal phase III VELOUR trial, which showed a significant improvement in overall survival, progression-free survival, and response rates compared with FOLFIRI alone in patients previously treated with oxaliplatin-based chemotherapy 15. However, it is important to note that the VELOUR trial did not specifically evaluate patients progressing after bevacizumab-based first-line therapy, and the proportion of patients previously exposed to bevacizumab was limited. Therefore, the applicability of these findings to a strictly post-bevacizumab population remains uncertain. In this context, our study, which exclusively included patients progressing after first-line FOLFOX plus bevacizumab, provides complementary real-world evidence suggesting that switching to aflibercept may not confer a significant survival advantage over continuing bevacizumab beyond progression.

Despite the strengths of this real-world analysis, several limitations should be acknowledged. First, its retrospective design may introduce selection bias and limit the ability to establish causal relationships between treatment and outcomes. Second, although all patients received a uniform first-line FOLFOX plus bevacizumab regimen, treatment allocation in the second-line setting was not randomized and was based on physician preference. Although stabilized IPTW achieved adequate balance across measured baseline covariates, residual confounding from unmeasured factors cannot be excluded. Third, because of the retrospective design, no a priori sample size calculation was performed. The relatively limited sample size may have reduced the ability to detect modest differences between treatment groups, and the possibility of a type II error cannot be excluded. Based on the event information and variance estimates from the robust IPTW-weighted Cox models, the approximate minimum detectable HRs at a two-sided α of 0.05 and 80% power were 1.62 for PFS and 1.67 for OS. These estimates indicate that smaller treatment effects may not have been reliably detected in the present study. Therefore, the nonsignificant findings should not be interpreted as evidence of equivalence between the two treatment strategies. Additionally, MSI status was unknown in a substantial proportion of patients; however, a sensitivity analysis restricted to patients with known MSI status yielded treatment-effect estimates consistent with the primary IPTW analysis. Molecular heterogeneity, including RAS and BRAF status, may also have influenced treatment outcomes.

Overall, our findings contribute to the growing body of real-world evidence evaluating anti-angiogenic strategies in the second-line treatment of metastatic colorectal cancer. In contrast to some previous studies suggesting a survival advantage with continued bevacizumab, our results indicate comparable outcomes between bevacizumab and aflibercept in a homogeneous post-bevacizumab population. These differences across studies likely reflect variations in patient selection, molecular characteristics, and study design, underscoring the complexity of treatment decision-making in this setting.

## Conclusion

FOLFIRI combined with either bevacizumab or aflibercept showed no statistically significant differences in survival outcomes in patients with metastatic colorectal cancer progressing after first-line FOLFOX plus bevacizumab. Both strategies may represent reasonable second-line options in this specific clinical setting. Further prospective studies are needed to better define the optimal sequencing of anti-angiogenic therapies.

## Figures and Tables

**Figure 1:**
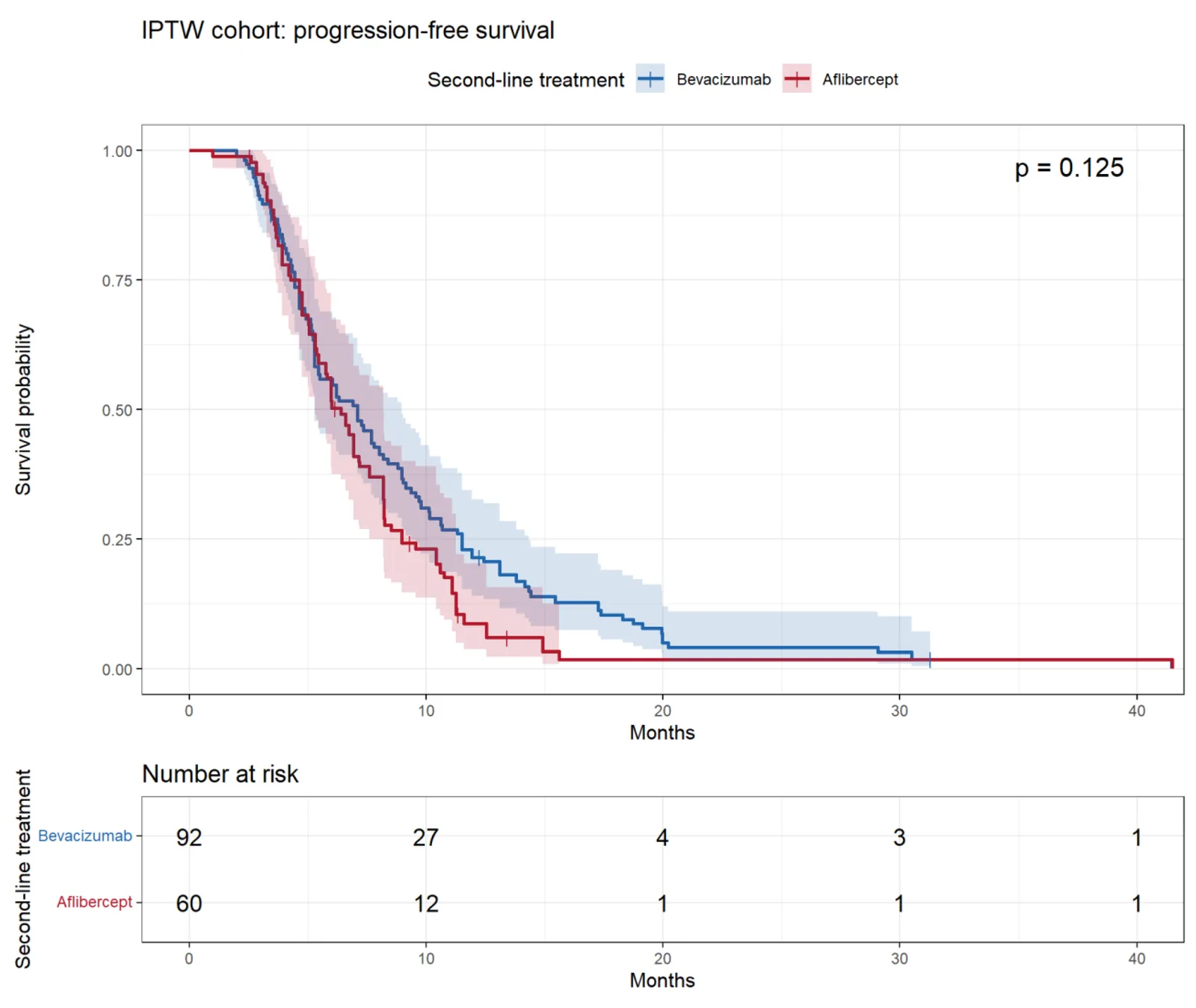
** IPTW-weighted Kaplan-Meier curves for progression-free survival (PFS) according to second-line treatment with FOLFIRI plus bevacizumab or aflibercept. Shaded areas represent 95% confidence intervals; censoring events are indicated by tick marks. Numbers at risk are shown below the plot.** PFS: progression-free survival.

**Figure 2:**
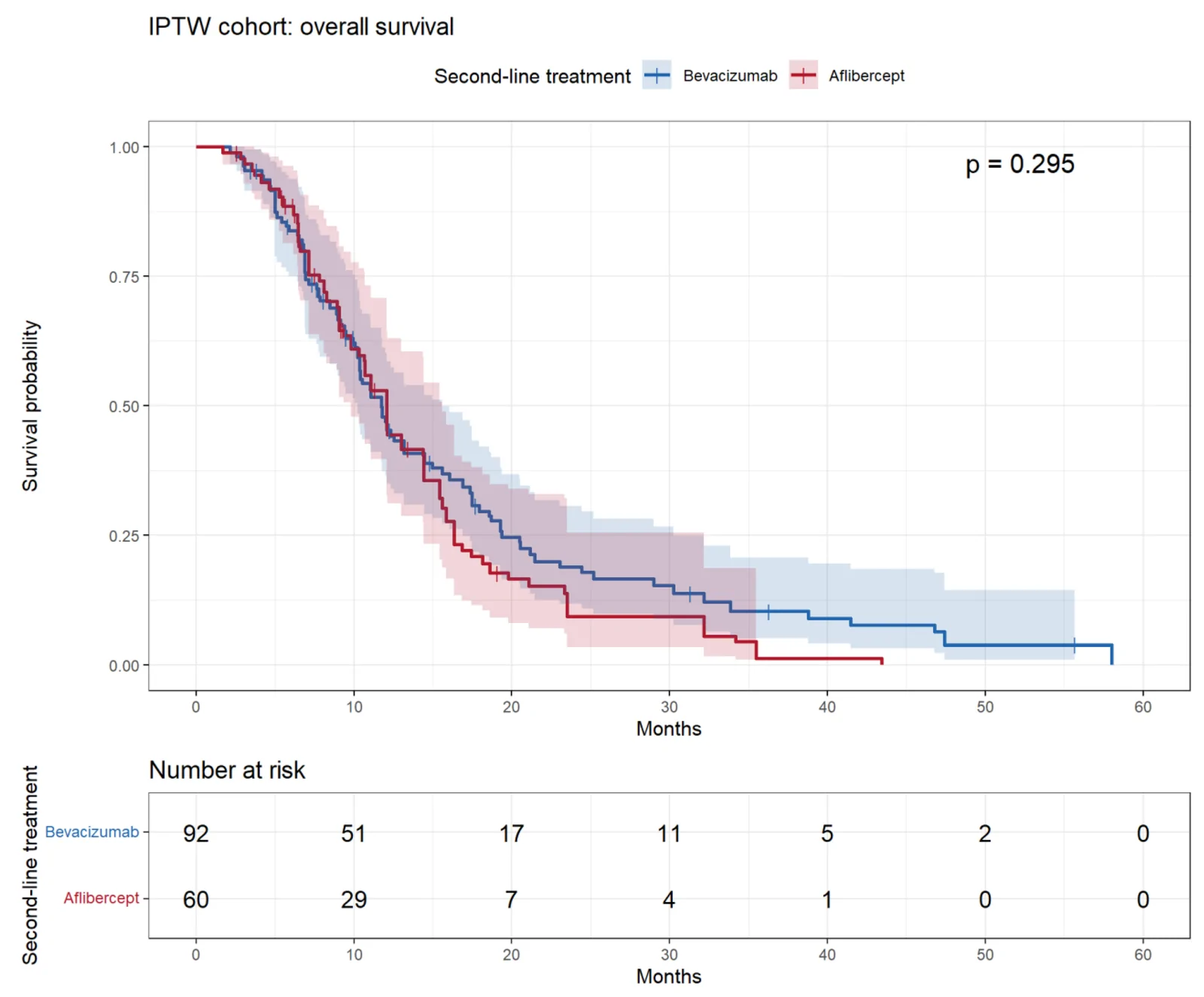
** IPTW-weighted Kaplan-Meier curves for overall survival (OS) according to second-line treatment with FOLFIRI plus bevacizumab or aflibercept. Shaded areas represent 95% confidence intervals; censoring events are indicated by tick marks. Numbers at risk are shown below the plot.** OS: overall survival.

**Table 1:** Baseline demographic and clinicopathological characteristics according to second-line treatment and covariate balance after inverse probability of treatment weighting (IPTW)

		FOLFIRI + Bevacizumab, n (%)	FOLFIRI + Aflibercept, n (%)	FOLFIRI + Bevacizumab, IPTW-weighted %	FOLFIRI + Aflibercept, IPTW-weighted %	Weighted p value	Max |SMD| after IPTW
Sex	Woman	39 (42.4)	22 (36.7)	40.6	42.6	0.835	0.020
	Man	53 (57.6)	38 (63.3)	59.4	57.4		
Age	< 65	59 (64.1)	39 (65.0)	62.0	65.7	0.682	0.037
	≥ 65	33 (35.9)	21 (35.0)	38.0	34.3		
ECOG	0	37 (40.2)	40 (66.7)	50.8	53.0	0.815	0.022
	1	55 (59.8)	20 (33.3)	49.2	47.0		
Comorbidity	Yes	51 (55.4)	34 (56.7)	59.6	60.2	0.946	0.006
	No	41 (44.6)	26 (43.3)	40.4	39.8		
Tumor location	Right-sided colon	27 (29.3)	14 (23.3)	28.4	28.7	0.962	0.022
	Left-sided colon	47 (51.1)	37 (61.7)	52.8	50.6		
	Rectum	18 (19.6)	9 (15.0)	18.7	20.7		
Histology	Adenocarcinoma	87 (94.6)	56 (93.3)	93.5	91.7	0.729	0.018
	Mucinous adenocarcinoma	5 (5.4)	4 (6.7)	6.5	8.3		
MSI status	MSS	67 (72.8)	31 (51.7)	67.7	66.9	—	0.009
	MSI-H	1 (1.1)	2 (3.3)	1.6	1.9		
	Unknown	24 (26.1)	27 (45.0)	30.7	31.2		
De novo metastasis	Yes	83 (90.2)	54 (90.0)	90.8	92.3	0.737	0.016
	No	9 (9.8)	6 (10.0)	9.2	7.7		
Resection status	R0	8 (8.7)	6 (10.0)	8.4	7.7	—	0.038
	R1	2 (2.2)	0	2.2	0.0		
	R2	21 (22.8)	11 (18.3)	23.6	22.7		
	Non-operated	61 (66.3)	43 (71.7)	65.8	69.6		
KRAS status	Wild-type	38 (41.3)	24 (40.0)	42.7	36.9	—	0.058
	Mutant	54 (58.7)	36 (60.0)	57.3	63.1		
NRAS status	Wild-type	88 (95.7)	57 (96.6)	96.4	93.5	—	0.029
	Mutant	4 (4.3)	2 (3.4)	3.6	4.4		
BRAF status	Wild-type	88 (95.7)	57 (96.6)	95.9	95.8	—	0.021
	Mutant	4 (4.3)	2 (3.4)	4.1	2.1		
Liver metastasis	Yes	76 (82.6)	55 (91.7)	86.0	84.3	0.810	0.017
	No	16 (17.4)	5 (8.3)	14.0	15.7		
Lung metastasis	Yes	22 (23.9)	20 (33.3)	29.7	37.0	0.442	0.072
	No	70 (76.1)	40 (66.7)	70.3	63.0		
Peritoneal metastasis	Yes	11 (12.0)	3 (5.0)	9.5	9.2	0.951	0.003
	No	81 (88.0)	57 (95.0)	90.5	90.8		
Bone metastasis	Yes	9 (9.8)	4 (6.7)	8.7	10.9	0.724	0.021
	No	83 (90.2)	56 (93.3)	91.3	89.1		
Non-regional lymph node metastasis	Yes	11 (12.0)	10 (16.7)	14.4	15.6	0.860	0.012
	No	81 (88.0)	50 (83.3)	85.6	84.4		
Local treatment for metastatic disease	Yes	15 (16.3)	7 (11.7)	16.8	11.6	0.431	0.053
	No	77 (83.7)	53 (88.3)	83.2	88.4		

ECOG, Eastern Cooperative Oncology Group; MSI, microsatellite instability; MSS, microsatellite stable; MSI-H, microsatellite instability-high; R0, complete resection; R1, microscopic residual disease; R2, macroscopic residual disease; IPTW, inverse probability of treatment weighting; SMD, standardized mean difference; Max |SMD|, maximum absolute standardized mean difference. For variables with multiple categories, Max |SMD| represents the maximum absolute SMD across category levels after IPTW.

**Table 2:** IPTW-weighted univariate and multivariate Cox regression analyses for progression-free survival (PFS)

Variables	Univariate analysis		Multivariate analysis	
	HR (95% CI)	p value	HR (95% CI)	p value
Sex Men (R) Women	1.145 (0.822-1.594)	0.423	1.144 (0.790-1.657)	0.475
Age <65 (R) ≥65	1.627 (1.158-2.286)	0.005	1.857 (1.226-2.812)	0.003
ECOG 0 (R) 1	1.039 (0.749-1.443)	0.818	0.916 (0.612-1.372)	0.671
Comorbidity No (R) Yes	0.952 (0.683-1.325)	0.769		
Tumor location Right-sided colon (R) Left-sided colon Rectum	0.830 (0.561-1.227) 0.810 (0.515-1.273)	0.350 0.361		
Tumor presentation De novo metastasis (R) Metachronous metastasis	0.846 (0.534-1.341)	0.478		
RAS/BRAF mutation status Wild-type (R) Mutant	1.163 (0.817-1.656)	0.401	1.142 (0.806-1.620)	0.455
Liver metastasis No (R) Yes	1.513 (1.012-2.263)	0.043	1.758 (1.176-2.629)	0.006
Lung metastasis No (R) Yes	1.024 (0.722-1.453)	0.895		
Peritoneal metastasis No (R) Yes	1.276 (0.696-2.340)	0.431		
Bone metastasis No (R) Yes	1.778 (1.100-2.875)	0.019	2.208 (1.276-3.820)	0.005
Non-regional lymph node metastasis No (R) Yes	0.814 (0.537-1.232)	0.330		
Any-grade toxicity No (R) Yes	1.129 (0.801-1.592)	0.488		
Grade 3-4 toxicity No (R) Yes	0.839 (0.588-1.197)	0.333		
Treatment discontinuation due to toxicity Yes (R) No	1.103 (0.789-1.540)	0.567		
Dose reduction due to toxicity Yes (R) No	1.021 (0.713-1.461)	0.911		
Treatment Bevacizumab (R) Aflibercept	1.302 (0.929-1.825)	0.125	1.347 (0.960-1.889)	0.085

ECOG: Eastern Cooperative Oncology Group

**Table 3:** IPTW-weighted univariate and multivariate Cox regression analyses for overall survival (OS)

Variables	Univariate analysis		Multivariate analysis	
	HR (95% CI)	p value	HR (95% CI)	p value
Sex Men (R) Women	1.090 (0.730-1.628)	0.672	1.009 (0.672-1.514)	0.966
Age <65 (R) ≥65	1.435 (0.989-2.082)	0.057	1.545 (0.941-2.536)	0.085
ECOG 0 (R) 1	1.093 (0.750-1.594)	0.644	0.976 (0.577-1.652)	0.929
Comorbidity No (R) Yes	1.021 (0.701-1.487)	0.912		
Tumor location Right-sided colon (R) Left-sided colon Rectum	0.833 (0.522-1.329) 0.814 (0.491-1.351)	0.443 0.426		
Tumor presentation De novo metastasis (R) Metachronous metastasis	0.870 (0.490-1.545)	0.635		
RAS/BRAF mutation status Wild-type (R) Mutant	0.976 (0.631-1.511)	0.914		
Liver metastasis No (R) Yes	1.342 (0.867-2.078)	0.211		
Lung metastasis No (R) Yes	0.978 (0.661-1.447)	0.912		
Peritoneal metastasis No (R) Yes	0.911 (0.419-1.979)	0.814		
Bone metastasis No (R) Yes	2.136 (1.225-3.726)	0.007	2.354 (1.291-4.294)	0.005
Non-regional lymph node metastasis No (R) Yes	0.892 (0.523-1.522)	0.676		
Any-grade toxicity No (R) Yes	1.041 (0.690-1.570)	0.847		
Grade 3-4 toxicity No (R) Yes	0.951 (0.642-1.407)	0.800		
Treatment discontinuation due to toxicity Yes (R) No	1.077 (0.736-1.578)	0.702		
Dose reduction due to toxicity Yes (R) No	0.825 (0.563-1.208)	0.323		
Treatment Bevacizumab (R) Aflibercept	1.212 (0.846-1.736)	0.295	1.122 (0.751-1.678)	0.573

ECOG: Eastern Cooperative Oncology Group

**Table 4:** Grade 3-4 treatment-related toxicities before and after inverse probability of treatment weighting (IPTW)

	FOLFIRI + Bevacizumab, (n=92), n (%)	FOLFIRI + Aflibercept, (n=60), n (%)	IPTW-weighted FOLFIRI + Bevacizumab, %	IPTW-weighted FOLFIRI + Aflibercept, %
Neutropenia	11 (12.0)	14 (23.3)	11.5	20.3
Anemia	4 (4.3)	4 (6.7)	3.9	5.3
Thrombocytopenia	3 (3.2)	10 (16.7)	3.3	15.6
Hepatotoxicity	2 (2.2)	0 (0)	2.2	0.0
Neuropathy	1 (1.1)	2 (3.3)	2.2	5.0
Nephrotoxicity	1 (1.1)	1 (1.7)	0.8	0.9
Diarrhea	1 (1.1)	1 (1.7)	1.2	0.9

## Data Availability

The datasets generated and analyzed during the current study are available from the corresponding author on reasonable request.

## Abbreviations

CRC: colorectal cancer
mCRC: metastatic colorectal cancer
PFS: progression-free survival
OS: overall survival
CI: confidence interval
HR: hazard ratio
ECOG: Eastern Cooperative Oncology Group
MSI: microsatellite instability
MSS: microsatellite stable
MSI-H: microsatellite instability-high
RAS: rat sarcoma viral oncogene homolog
KRAS: Kirsten rat sarcoma viral oncogene homolog
NRAS: neuroblastoma rat sarcoma viral oncogene homolog
BRAF: B-Raf proto-oncogene
VEGF: vascular endothelial growth factor
EGFR: epidermal growth factor receptor
IPTW: inverse probability of treatment weighting
ATE: average treatment effect
